# Critical Aspects of Various Techniques for Synthesizing Metal Oxides and Fabricating Their Composite-Based Supercapacitor Electrodes: A Review

**DOI:** 10.3390/nano12111873

**Published:** 2022-05-30

**Authors:** Mohd Zahid Ansari, Kang-Min Seo, Soo-Hyun Kim, Sajid Ali Ansari

**Affiliations:** 1 School of Materials Science and Engineering, Yeungnam University, Gyeongsan 38541, Korea; zahid.smr@yu.ac.kr (M.Z.A.); skm1274@naver.com (K.-M.S.); 2 Institute of Materials Technology, Yeungnam University, Gyeongsan 38541, Korea; 3 Department of Physics, College of Science, King Faisal University, P.O. Box 400, Hofuf 31982, Saudi Arabia; sansari@kfu.edu.sa

**Keywords:** energy storage devices, supercapacitors, metal oxides, electrode materials, synthesis techniques

## Abstract

Supercapacitors (SCs) have attracted attention as an important energy source for various applications owing to their high power outputs and outstanding energy densities. The electrochemical performance of an SC device is predominantly determined by electrode materials, and thus, the selection and synthesis of the materials are crucial. Metal oxides (MOs) and their composites are the most widely used pseudocapacitive SC electrode materials. The basic requirements for fabricating high-performance SC electrodes include synthesizing and/or chemically modifying unique conducting nanostructures, optimizing a heterostructure morphology, and generating large-surface-area electroactive sites, all of which predominantly rely on various techniques used for synthesizing MO materials and fabricating MO- and MO-composite-based SC electrodes. Therefore, an SC’s background and critical aspects, the challenges associated with the predominant synthesis techniques (including hydrothermal and microwave-assisted syntheses and chemical-bath and atomic-layer depositions), and resulting electrode electrochemical performances should be summarized in a convenient, accessible report to accelerate the development of materials for industrial SC applications. Therefore, we reviewed the most pertinent studies on these synthesis techniques to provide insight into the most recent advances in synthesizing MOs and fabricating their composite-based SC electrodes as well as to propose research directions for developing MO-based electrodes for applications to next-generation SCs.

## 1. Introduction

**Figure 1:**
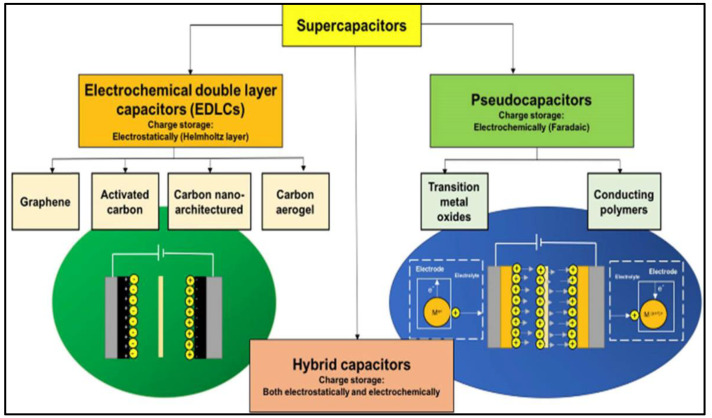
Schematic classifying different SC types and their underlying charge-storage mechanism. Reprinted from Ref. [13].

The requirement of using energy-storage devices for multifunctional applications is currently a hot research topic. Technological advancements ranging from electric vehicles to miniature portable electronics have considerably increased the demand for energy storage devices. Electrochemical energy storage is the most effective, cheap, and convenient method for meeting this demand [1,2]. Owing to their low costs and versatile performance, batteries and electrochemical capacitors (ECs) are two important categories of electrochemical energy-storage devices [3]. Although batteries are well known for exhibiting high energy densities, their lifespans are short and they exhibit low power densities [4,5,6,7]. In contrast, supercapacitors (SCs) are the most promising electrochemical energy storage devices that bridge the gap between conventional capacitors and batteries. SCs are notable for their unique high power densities, wide working-temperature ranges, extended cycling lives, improved energy densities compared with traditional capacitors, low maintenance, low leakage currents, long service lives, and high efficiencies [8,9,10]. Hence, SCs are employed in a wide range of applications, such as portable electronics, regenerative power-braking systems, hybrid electric vehicles, spacecrafts, aircrafts, military devices, biomedical equipment, and wind-energy power generators [11,12]. As a result, SCs have attracted considerable attention from both research and technological perspectives. However, SCs exhibit low energy densities, which are major drawbacks that limit their use in applications requiring both high energy and power densities. SCs have been broadly categorized as electric double-layer capacitors (EDLCs) and pseudocapacitors (PCs) according to their charge storage mechanisms, as shown in Figure 1 [13]. 

**Scheme 1:**
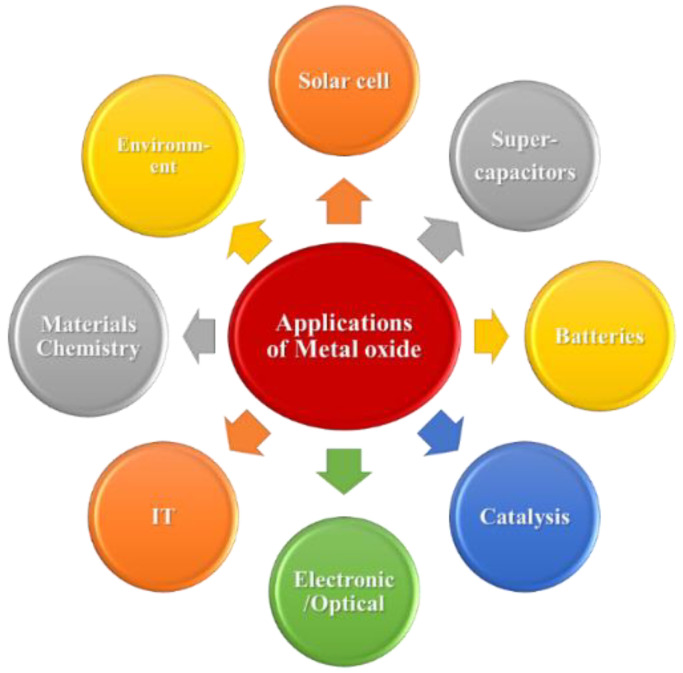
Different MO material applications.

EDLCs are so named because they physically store an electrostatic charge in an electric field between an adjacent electrode and electrolyte, and the charge rapidly accumulates through electrostatic attraction without any chemical reactions, which generates a high device-power density [13,14,15]. In contrast, PCs operate by faradaically storing a charge at the electrode–electrolyte interface, where the charge is actually transferred between the electrode and the electrolyte. Through reversible redox reactions involving the highly reversible formation of chemical species, PCs exhibit higher energy densities than EDLCs [16,17,18]. The main components required to fabricate a hybrid-operating SC device include electrode materials, electrolytes, separators, and current collectors [19]. Although each component is important, selecting an electrode’s material and fabricating the electrode are the most crucial aspects. Carbon-based materials are the best EDLC electrode materials because their charge-storage mechanism mainly relies on the electrode-material surface area accessible to electrolyte ions. Activated carbon (AC), graphene and its derivatives, carbon nanotubes (CNTs), and carbon aerogels have been used as EDLC electrode materials [20,21,22] because they are inexpensive, are simply fabricated, and exhibit large surface areas and wide ranges of pore-size distributions. However, owing to their low specific capacitances, carbonaceous materials do not sufficiently contribute to the high SC energy density [23]. High-capacitance electrode materials are preferentially used to overcome the low energy density. Owing to their rapid reversible surface-redox reactions, conducting polymers and metal oxides (MOs) are pseudocapacitive electrode materials that provide high energy densities to SCs [24,25,26]. Compared to MOs, conducting polymers exhibit poor structural stabilities during continuous charge–discharge cycling. Therefore, various low-cost MOs and their composite electrode materials have been identified as promising alternatives to carbonaceous materials and conducting polymers [27,28,29]. Because of their exceptional physicochemical properties, MOs have been used in several applications (Scheme 1) and are usually applied as electrode materials because they can be synthesized using a wide range of methods and because of their high theoretical specific capacitances, natural abundance, excellent pseudocapacitive behaviors, high thermal and electrochemical stabilities, and reliabilities [30,31,32,33].

**Scheme 2:**
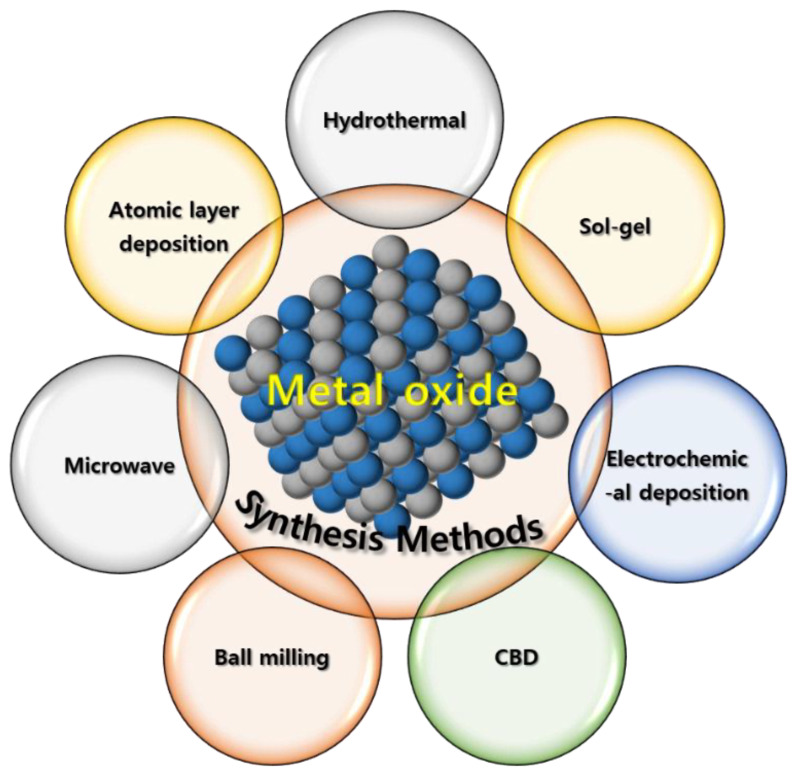
Different techniques for preparing MO materials.

MO-based SC electrodes have been synthesized using various chemical and vapor-phase-based techniques, including hydrothermal synthesis [34], sol-gel processing [35], electrochemical/electropolymerization synthesis [36], chemical bath deposition (CBD) [37], solvothermal synthesis [38], microwave-assisted synthesis [39], atomic-layer deposition (ALD) [40], and ball milling [41] (Scheme 2). However, not all these techniques can be applied to industrially produce high-quality low-cost MO-based SC electrodes. Therefore, an easy, efficient, economic, nontoxic, and reliable technique that can be used to produce numerous high-purity materials under mild reaction conditions should be developed for fabricating MO-based SC electrodes [42,43,44,45].

The primary goal of this review was to provide a thorough understanding of hydrothermal and microwave-assisted syntheses and chemical-bath and atomic-layer depositions, which are currently the techniques predominantly used for preparing MO-based SC electrodes, to explore MO-based materials’ supercapacitive performance.

For all the synthesis techniques, especially from the most frequently used method to emerging deposition methods, such as hydrothermal synthesis, microwave-assisted synthesis, chemical-bath deposition, and atomic-layer deposition, reviewed herein, we first discuss the fundamentals, including the principle and experimental setup, and provide a schematic representation. The latest studies are then reviewed to compile the critical aspects of each synthesis technique and evaluate the electrochemical performance of the materials prepared using the technique. The electrode materials produced using each synthesis technique are emphasized. Furthermore, the challenges encountered in previous research and how the challenges are being addressed using the currently predominant synthesis techniques are explored and highlighted. This article is divided into six main sections, including the Introduction. Section 2 discusses hydrothermal synthesis, its critical aspects, and its application for preparing MO-based SC electrodes. Section 3 discusses microwave-assisted synthesis and its importance and challenges in preparing MO-based SC electrodes. Section 4 focuses on CBD and its application for preparing MO-based SC electrodes. Section 5 describes the ALD method for producing MO-based SC electrodes, and Section 6 summarizes the critical electrode-material synthesis aspects and concludes with a brief research outlook.

## 2. Hydrothermal Synthesis

**Figure 2:**
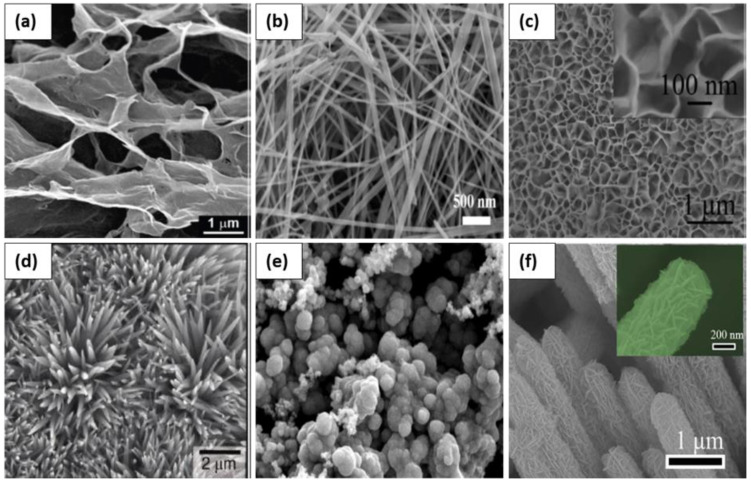
SEM images of (**a**) hydrothermally synthesized self-assembled graphene hydrogel (SGH) (reprinted with permission from Ref. [50]. Copyright 2010 American Chemical Society), (**b**) nanowire-like α-MnO_2_ (reprinted with permission from Ref. [51]. Copyright 2016 Elsevier), (**c**) flake-like mesoporous NiO (reprinted with permission from Ref. [52]. Copyright 2014 Elsevier), (**d**) Co_3_O_4_ nanostructures (reprinted with permission from Ref. [53]. Copyright 2011 American Chemical Society), (**e**) sphere-like MoO_2_ nanostructure (reprinted with permission from ref. [54]. Copyright 2016 Elsevier), and (**f**) hierarchical core–shell NiCo_2_O_4_@NiMoO_4_ nanowires hydrothermally grown on carbon cloth (reprinted with permission from Ref. [55]. Copyright 2015 Springer Nature).

Hydrothermal synthesis is the most widely used solution-based technique for fabricating SC electrodes; it enables electrode materials exhibiting one-, two-, and three-dimensional and hierarchical nanoarchitectures and well-defined morphologies to be fabricated [46]. Moreover, hydrothermal synthesis facilitates chemical reactions in aqueous media at desired pressures and temperatures [47,48]; chemicals are synthesized above the water boiling point, and water serves as an aqueous medium. With an increasing temperature, pressure begins to increase inside a sealed chamber and consequently initiates a thermochemical reaction. Materials are hydrothermally synthesized in a Teflon^TM^-lined stainless-steel autoclave [49], which is then placed in an oven and maintained at appropriate temperatures. Hydrothermal synthesis is simple and often referred to as a “environmentally friendly” because it promotes a low chemical consumption and does not emit any toxic gases. Moreover, hydrothermal synthesis is inexpensive, simple to use, requires few experimental conditions, and helps generate a wide range of materials for electrodes exhibiting well-defined morphologies (Figure 2a–f) [50,51,52,53,54,55]. In this section, the latest advancements made in hydrothermally synthesizing various MOs, such as ruthenium oxide, iron oxide, titanium oxide, and magnesium oxide, are discussed [56,57,58,59]. Additionally, the importance and challenges, latest findings, and critical aspects of hydrothermal synthesis are considered. Ruthenium oxide (RuO_2_) exhibits a high theoretical specific capacitance, wide operational potential window, good redox behavior, and long cycle life [60]. Some researchers [61,62] have hydrothermally synthesized RuO_2_ for applications in SC electrode materials and have investigated various methods for improving the material’s properties to increase the RuO_2_ SC-electrode specific capacitance, energy, and power density [63]. For instance, to hydrothermally synthesize electrode materials, a surfactant-assisted approach is highly desirable, and hydrothermal synthesis produces high-purity materials and enables the morphology and the particle size, nucleation, and growth to be precisely controlled [64]. For example, Vijaybala et al. used polyethylene glycol (PEG) as a surfactant to synthesize RuO_2_ nanosheets and studied the relative RuO_2_ nanosheet electrochemical performance. An electrode scanned at 5 mV s^−1^ exhibited a specific capacitance of 600 F g^−1^ [65].

**Figure 3:**
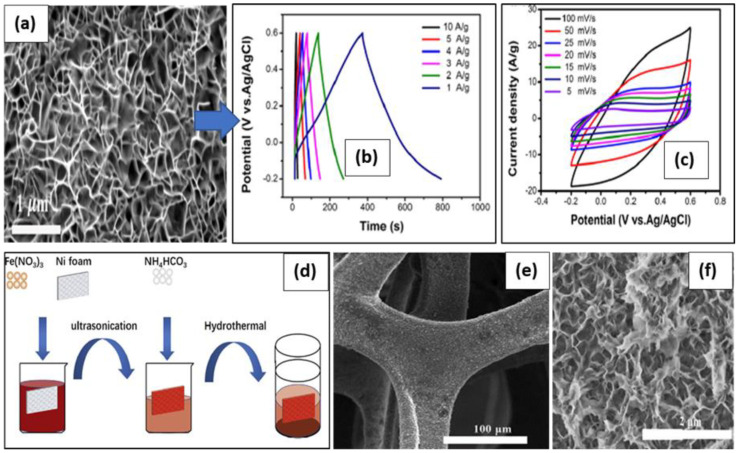
(**a**) SEM image and (**b**,**c**) electrochemical performance of porous MnO_2_ ultrathin nanosheets (reprinted with permission from Ref. [81]. Copyright 2021 Elsevier). (**d**) Schematic illustrating preparation and (**e**) magnified and (**f**) non-magnified SEM images of porous FeOOH hydrothermally synthesized directly on Ni foam (reprinted with permission from Ref. [93]. Copyright 2018 Elsevier).

Numerous studies have reported that porous materials with large surface areas are very promising for improving the electrode’s electrochemical performance [66,67]. Consequently, hard templates, such as silica and polymers, can be used to directly synthesize hollow-structured materials. Because hydrothermal synthesis enables the utilization of hard templates, Peng et al. recently used SiO_2_ as a hard template to synthesize a RuO_2_ honeycomb-like structure exhibiting a hollow spherical morphology for applications to SC electrodes [68]. Fabricated RHC and RHS electrodes exhibited specific capacitances of 628 and 597 F g^−1^ at a current density of 20 A g^−1^ and high stabilities with 86 and 91% capacitance retentions after 4000 continuous cycles, respectively. Despite being a potential electrode material for applications in energy storage devices, pristine RuO_2_ is expensive to produce, hydrous RuO_2_ is unstable, and RuO_2_ nanoparticles agglomerate during charge–discharge cycling. To overcome these limitations, other promising materials, such as carbonaceous materials, polymers, and MOs, have been incorporated with RuO_2_ to enhance the electrochemical performance of the electrode. Following the same approach, Hossain et al. fabricated a ruthenium–ruthenium oxide–AC (Ru–RuO_2_/AC) composite SC electrode and measured its electrochemical performance. At an optimized nanocomposite composition of 10 wt.% Ru–RuO_2_ and 90 wt.% AC, the electrode exhibited a high specific capacitance of 1460 F g^−1^ at a correspondingly high current density of 10 A g^−1^ in a 0.5 M H_2_SO_4_ electrolyte. Moreover, a high capacitance retention of 94% after 10,000 cycles was attributed to the uniform distribution of Ru–RuO_2_ nanoparticles in the AC, large specific surface area, and good material conductivity [69]. RuO_2_ has also been blended with other inexpensive transition-metal oxides (TMOs) to combine the redox activity of both merged components and develop an effective method for improving the electrode electrochemical performance while reducing the high RuO_2_ cost [70]. Yi et al. hydrothermally synthesized a graphene/RuO_2_/Co_3_O_4_ ternary nanocomposite as an SC electrode material, which both electrostatically and pseudocapacitively stored a charge to yield a high capacitance of 715 F g^−1^ at a current density of 1 A g^−1^ and exhibited an enhanced potential window of 1.4 V. The outstanding performance was attributed to a porous structure consisting of RuO_2_ and Co_3_O_4_ nanoparticles (diameter < 5 nm) uniformly distributed on graphene sheets [71]. Hu et al. fabricated RuO_2_·*x*H_2_O–TiO_2_ nanocomposite SC electrodes using advanced microwave-assisted hydrothermal techniques. An electrode fabricated using a composite containing 60% RuO_2_ and scanned at 100 mV s^−1^ exhibited a high specific capacitance of 992 F g^−1^. The considerably increased electrochemical performance was attributed to both the nanocomposite stability and unique microstructure [72]. Manganese is a multivalent transition metal abundant in Earth’s crust and forms various oxides, including MnO_2_, Mn_2_O_3_, Mn_3_O_4_, and MnO [73], which are distinguished by their crystal structures, defect chemistries, and unique morphologies, all of which make Mn an excellent SC electrode material [74,75]. Because of their excellent pseudocapacitance, high specific capacitance, abundance, and environmental friendliness, manganese oxides are potential next-generation materials for applications in SC electrodes and have even been substituted for RuO_2_-based electrode materials [76]. The chemical structure, surface morphology, crystallite size, pore volume, porosity, homogeneity, and conductivity of MnO_2_ are all important for fabricating electrodes exhibiting a superior electrochemical performance, and hydrothermal synthesis is particularly well suited for fabricating manganese-based inorganic SC-electrode materials. Furthermore, hydrothermal synthesis provides a well-suited environment and desirable temperature and pressure ranges for synthesizing uniform high-porosity high-purity electrode materials. Additionally, considerable progress has been made in hydrothermally synthesizing MnO_2_-based SC electrode materials [77,78,79]. For instance, Li et al. hydrothermally synthesized belt-like MnO_2_ nanomaterials exhibiting high porosities, large specific surface areas, and high conductivities [80]. Moreover, their method is helpful for engineering defects in the electrode material’s internal structure to increase the number of electroactive sites available for redox reactions and improve the electrode’s electrochemical behavior. Zhao et al. hydrothermally synthesized and subsequently thermally annealed MnO_2_ ultrathin nanosheets for different time periods to prepare binder-free electrode materials containing different oxygen defect concentrations, which directly affected the electrode’s electrochemical performance, as shown in Figure 3a–c. The electrode was annealed at 300 °C for 45 min and exhibited the highest specific capacitance of 522.5 F g^−1^ at a current density of 1 A g^−1^, and an asymmetrically configured device fabricated using this electrode exhibited an energy density of 18.06 W h kg^−1^ at a power density of 1 kW kg^−1^ in a 0–2V window [81].

Although MnO_2_ nanomaterials are promising candidates for applications in SC electrodes, these materials still exhibit low conductivities, which negatively impact the electrochemical performance of electrodes during cycling and limits the commercial production of such electrodes. Therefore, Mn-based composites must be synthesized to exploit the electrochemical synergy of both the components and enhance the conductivity. Carbonaceous materials, or conducting polymers, are used mostly for fabricating hybrid MnO_2_ electrodes. However, carbonaceous-material-based MnO_2_ nanocomposites are more important owing to their large specific surface areas, high conductivities, and electric double-layer capacitances. For instance, Ning et al. hydrothermally coated MnO_2_ nanosheets with electrospun carbon nanofibers to synthesize a carbon nanofiber/MnO_2_ nanosheet composite. Owing to a unique composite hollow structure, an electrode fabricated using the composite electrode exhibited a specific capacitance of 151 F g^−1^ at a current density of 1 A g^−1^ and capacitance retention of 90% after 8000 cycles [82]. The binder-free electrode improved the overall electrochemical performance of the SC device because the electrode reduced internal resistance. Hydrothermal synthesis enabled the economic and convenient fabrication of the binder-free electrode, and the electrode facilitated both a rapid electron transfer and electrolyte penetration deep into the active material pores. Similarly, Huang et al. hydrothermally synthesized a 3D CNT@MnO_2_ hybrid binder-free electrode in a facile manner; it exhibited a capacitance of 325.5 F g^−1^ at a current density of 0.3 A g^−1^ and a capacitance retention of 90.5% after 5000 cycles [83]. Flexible SCs have recently attracted considerable research attention because of their widespread applications in foldable displays, wearable electronics, and other miniature portable devices. Flexible materials must exhibit high mechanical strengths and a good electrochemical performance for applications to SC electrodes [84]. In 2016, Guo et al. hydrothermally synthesized a MnO_2_/graphene/polyester composite in a facile manner and used it to fabricate an SC electrode. Interestingly, the MnO_2_ morphology was easily controlled by maintaining a constant reaction time. When swept at 2 mV s^−1^, the electrode exhibited a specific capacitance of 332 F g^−1^ [85]. All these studies indicate that hydrothermal synthesis is an easy and convenient method for fabricating manganese-based SC electrodes. Among the transition metals, iron is the most promising for applications in energy-storage devices. Owing to their improved electrochemical performance in the negative potential window, iron oxides, hydroxides, and composites have been widely investigated for fabricating SC anodes [86,87]. Furthermore, because iron is naturally abundant, inexpensive, and nontoxic, it has attracted considerable research attention for applications in SC electrodes. Moreover, iron exhibits different oxidation states because it can form many oxides, including FeO, Fe_2_O_3_, FeOOH, Fe_3_O_4_, and Fe(OH)_3_, the most stable of which are Fe_3_O_4_, FeOOH, and Fe_2_O_3_ [88]. In aqueous solutions, iron exists as Fe^2+^ and Fe^3+^ ions, which are highly desirable for a good electrochemical performance. Because these materials are primarily distinguished by their crystal dimensions, surface characteristics, defect chemistries, morphologies, and textures, research should be conducted to determine how these properties impact the development of rationally designed SC electrode materials to enhance the electrode’s electrochemical performance. To optimize SC electrodes, a novel synthesis method is required, and hydrothermal synthesis is the most effective method for preparing 0-, 1-, and 2-D and other promising iron oxide/hydroxide hierarchical structures [89,90]. Zhu et al. hydrothermally synthesized Fe_2_O_3_ nanoparticles for applications in SC electrodes and investigated how precursor conditions, surfactants, stabilizing agents, and reaction times affected the material electrochemical performance. The electrode material exhibited a specific capacitance of 340.5 F g^−1^ at a current density of 1 A g^−1^ [91]. Structurally stable electrode materials exhibit a good electrochemical performance during continuous charge–discharge cycling, and amorphous materials exhibiting disordered structures have proven to be advantageous when utilized as SC electrodes. Hydrothermal synthesis is also suitable for preparing amorphous porous SC electrode materials [92]. For instance, Yang et al. recently utilized one-pot hydrothermal synthesis to directly grow amorphous FeOOH on nickel foam, as shown in Figure 3d–f. The optimized electrode fabricated using the FeOOH grown at 160 °C exhibited a high specific capacitance of 1300 F g^−1^ at a current density of 2 A g^−1^. 

Additionally, the 3D porous electrode retained 91% of its initial capacitance after 2000 charge–discharge cycles [93].

Chen et al. hydrothermally synthesized Fe_3_O_4_ cubes in a facile manner and used them to fabricate an electrode exhibiting a specific capacitance of 118.2 F g^−1^ at a current density of 6 mA g^−1^ in a 1 M Na_2_SO_4_ electrolyte solution. After 500 charge–discharge cycles, the electrode retained only 88.7% of its initial capacitance. The rapidly degrading cycling stability and low capacitive performance were major obstacles in the practical application of the electrode [94]. To overcome these obstacles, researchers have been developing chemically modified electrode materials and incorporating other highly conducting components. For example, Yang et al. used this strategy to hydrothermally synthesize Fe_2_O_3_/graphene nanocomposites. At the optimized temperature, the FeOOH phase transitioned to Fe_2_O_3_. In a 1 M aqueous Na_2_SO_4_ electrolyte, the electrode exhibited a high capacitance of 343.7 F g^−1^ at a current density of 3 A g^−1^. The electrochemical performance of the electrode was attributed to synergistic contributions from the Fe_2_O_3_ mesoporous crystal structure and crumpled graphene sheets [95]. Although iron oxides/hydroxides must be extrinsically modified, intrinsically modifying them by generating oxygen vacancies, controlling their crystallinities, doping them with foreign ions, and modifying their crystal structures are equally important [96]. Wang et al. used an environmentally friendly hydrothermal synthesis method to fabricate a nitrogen/sulfur dual-doped N–Fe_2_O_3_-composite SC electrode, which exhibited a high-rate performance by retaining 91.3% of its initial capacitance after 5000 cycles at a current density of 5 A g^−1^. Furthermore, in a 6 M KOH electrolyte, the electrode exhibited a high specific capacitance of 156 F g^−1^ at a current density of 10 A g^−1^. This electrochemical behavior was attributed to a doping of the graphene matrix with nitrogen and sulfur, which introduced more defects and generated numerous electroactive sites [97].

Currently, materials based on metal organic frameworks (MOFs) are considered an important class of materials for a wide range of applications, including supercapacitors [98,99]. Recently, Chhetri et al. synthesized Cu_x_O-C/PANI by using the in situ polymerization of aniline on multilayered mesoporous Cu_x_O-C from Cu-MOF, which could provide a potential route for electron transfer and improve the kinetics of redox reactions. In the electrochemical response, the Cu_x_O-C/PANI electrode showed a high specific capacitance value (1308 F g^−1^ at 1 A g^−1^) with an 88.3% capacitance retention after a long-term charge-discharge process (10,000 cycles). Moreover, as a fabricated device using Cu_x_O-C/PANI//ZIF-8NPC electrodeshowed the highest energy density value of 55.1 W h kg^−1^ (at a 862.4 W kg^−1^ power density) and a power density of 8668.2 W kg^−1^ (at a 30.1 W h kg^−1^ energy density). As-fabricated MOF-derived CuxO@C/PANI electrodes for supercapacitors, which are decorated with PANI fibers, not only have improved conductivities but also serve as the interfaces between Cu_x_O/C and current collectors, providing an improved electrochemical performance [99]. These studies all focused on the advantages of hydrothermally synthesizing MOs and fabricating their composite-based SC electrodes. An ease of use and the ability to inexpensively synthesize multidimensional morphologies and high-purity crystalline nanostructures make hydrothermal synthesis unique. Additionally, hydrothermal synthesis is environmentally friendly because hazardous catalysts are not used and toxic gases cannot escape because a chamber used in the process is sealed. More importantly, hydrothermal synthesis can be combined with other synthesis methods, such as ultrasonication, chemical vapor deposition, microwave-assisted synthesis, and thermal reduction, to prepare advanced electrode materials [98,99,100]. However, a long reaction time is a major drawback of hydrothermal synthesis.

## 3. Microwave-Assisted Synthesis

**Figure 4:**
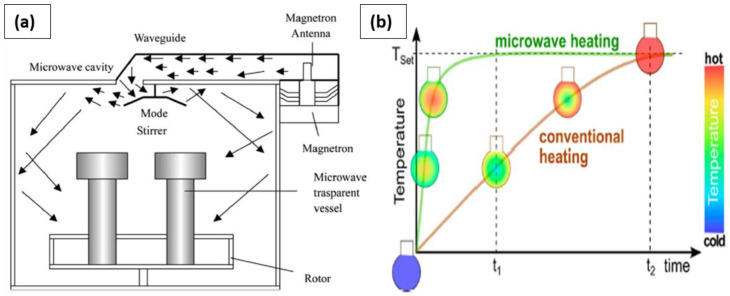
(**a**) Schematic representing microwave equipment and all components required for synthesizing inorganic materials (reproduced with permission from Ref. [108]. Copyright 2014 Elsevier). (**b**) Graph representing relationship between temperature and time for microwave-assisted and conventional heating methods (reproduced with permission from Ref. [101]. Copyright 2018 Taylor & Francis).

Microwave-assisted synthesis is a novel and appropriate technique for synthesizing the inorganic materials used in various industrial applications and is a rapidly growing research area. Microwaves have a wide range of applications, including food preparation, mining, rubber and plastic treatment, and wood drying [101,102,103]. Microwave-assisted synthesis can be used to rapidly, easily, inexpensively, uniformly, and efficiently heat materials. Compared to the conventional heating methods used to synthesize porous materials, organic and inorganic compounds, and nanocrystalline materials, microwave-assisted synthesis is advantageous for preparing MOs and fabricating their composite-based SC electrodes because it produces various multidimensional morphologies and crystal structures [104,105,106]. In conventional material heating methods, thermal energy is transferred convectively to a material’s surface and conductively to the material’s bulk. Microwaves are nonionizing electromagnetic radiation exhibiting wavelengths between those of radio and infrared waves and frequencies ranging from 0.3 to 300 MHz. Microwaves convert electromagnetic energy into thermal energy [107]. An electromagnetic field heats material through molecular interactions, and microwaves penetrate the material to provide energy, which entails dielectric heating through a dipole rotation and resonance energy absorption. Because the heat is molecularly transferred, the material is uniformly heated throughout its volume. Therefore, when microwave energy interacts with different dielectric materials, it selectively couples with higher-loss-tangent materials. A microwave heating instrument comprises six main components that generate microwaves. As shown in Figure 4a [108], these components include microwave generators (i.e., magnetrons), mode stirrers, turntables, circulators, waveguides, and microwave cavities. Because materials are superheated by microwaves in closed reaction chambers, the materials quickly reach the temperatures required for initiating chemical reactions. Figure 4b [101] shows that microwave-heated material rapidly reaches a reaction temperature compared to a conventionally heated counterpart. MOs and their composites exhibit high specific capacitances and energy densities. Using microwave heating to synthesize these materials is an efficient method for fabricating SC electrodes exhibiting unique morphologies, uniform pore-size distributions, different multidimensional nanostructures, and high mechanical and chemical stabilities [109,110]. Irradiating precursor materials with microwaves to molecularly heat the precursors and synthesize MOs and their composites accelerates the crystallization kinetics, rapidly and uniformly heats the precursors to the desired temperatures, forms unique metastable phases, increases energy efficiency, and reduces costs. This section focuses on the critical aspects of using microwave-assisted synthesis to prepare MOs and subsequently fabricate their composite-based SC electrodes.

**Figure 5:**
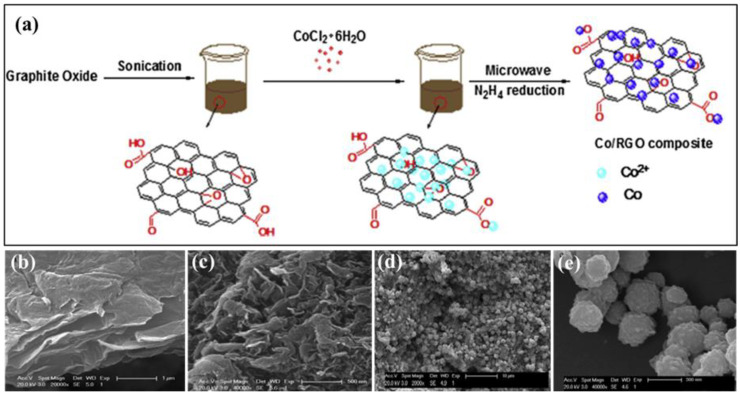
(**a**) Schematic illustrating formation mechanism of Co/rGO composite synthesized using microwave heating. SEM images of (**b**) GO, (**c**) rGO, (**d**) Co, and (**e**) Co/rGO-15 at different magnifications (reprinted with permission from Ref. [140]. Copyright 2015 Elsevier).

Microwave-assisted synthesis is highly suitable for preparing TMOs such as nickel oxides, cobalt oxides, vanadium oxides, and their composites [108]. Among the various TMOs, nickel oxides and hydroxides are excellent pseudocapacitive SC electrode materials. Owing to its large specific surface area, NiO is well known for its high theoretical specific capacitance (2573 F g^−1^). Moreover, NiO exhibits a high porosity in nanocrystal structures [111]. Furthermore, nickel oxides are promising SC electrode materials because nickel oxides are inexpensive, chemically stable, and nontoxic and can exhibit different morphologies [112,113], including nanosheets [114], nanoflakes [115], nano/microspheres [116], and nanotubes [117]. Although several methods, including hydrothermal [118] and sol–gel [119] syntheses and CBD [120], have been used to develop materials exhibiting such morphologies, microwave-assisted synthesis has proven to be an easier and more effective method [121,122]. In practice, microwave-assisted synthesis accelerates reaction kinetics and enables the rapid formation of numerous nanostructures. Although microwave-assisted synthesis operates based on electromagnetic radiation, the radiation itself does not ionize and remains inert while SC electrodes are being fabricated. In microwave-assisted synthesis, materials are heated through ionic conduction and dipolar polarization. Several researchers have used microwave-assisted synthesis to fabricate NiO electrode materials. For instance, Meher et al. extensively used microwave-assisted synthesis to prepare porous NiO and found that the NiO prepared using microwave-assisted synthesis exhibited an electrochemical performance superior to that of NiO prepared using conventional refluxing. Flake-like and porous sphere-like NiO materials obtained using conventional refluxing and microwave-assisted synthesis at 120 °C for 12 h and 15 min exhibited specific capacitances of 101 and 370 F g^−1^ at discharge current densities of 2 A g^−1^, respectively. The electrode electrochemical performance was enhanced because the microwave-synthesized NiO generated electroactive sites on the ripple-shaped nanospherical surface, which enabled the electrolyte to smoothly penetrate the electrode [123]. The SC electrode’s electrochemical performance mainly depends on the specific surface area of the electrode, the presence of electroactive sites, and morphology. Nickel oxides have been extensively studied to fabricate electrodes exhibiting such properties, and hollow nanospheres have several advantages over other morphologies. For example, owing to their hollow interior space, hollow nanospheres exhibit a reduced mass, which increases both spatial dispersion and effective mass transport. Microwave-assisted synthesis can be effectively applied to prepare materials exhibiting such morphologies. For instance, Cao et al. synthesized flower-like NiO hollow nanospheres. The precursor material was placed in a Teflon^TM^-lined autoclave and irradiated with microwaves at 170 °C for 3 min and was subsequently annealed at 300 °C to convert the flower-like hollow nanospheres into hollow NiO nanospheres. The electrodes rapidly fabricated using microwave-assisted synthesis exhibited an enhanced specific capacitance of 585 F g^−1^ at a high current density of 5 A g^−1^ [124]. Because of their excellent electrochemical performance, metal–organic frameworks (MOFs) have recently attracted considerable research interest for fabricating SC electrodes. Owing to its rapid reaction, uniform volumetric heating, and effective energy reduction, microwave-assisted heating was far superior to conventional heating for synthesizing MOFs. Recently, Han et al. irradiated pillared Ni–MOF (Ni(bdc)(ted)_0.5_) with microwaves for 30 min to synthesize hierarchical NiO nanoparticles. A NiO-350 SC electrode thermally annealed at the optimized temperature exhibited a specific capacitance of 248.28 F g^−1^ at a current density of 0.5 A g^−1^ and a cycling stability of 74.3% after 2000 cycles. The superior electrochemical performance of the electrode was attributed to the improved electrolyte ion transport deep in the porous electrode’s inner surface [125]. In theory, microwave-assisted synthesis could be exclusively used to fabricate MOs and their composites because it is simple, fast, inexpensive, and energy efficient and because it produces highly uniform materials. However, in practice, exclusively using microwave-assisted synthesis to control an MO and composite morphologies remains challenging [112,126]. Therefore, microwave-assisted hydrothermal synthesis has been universally adopted for preparing MOs and their composites because it exhibits rapid crystallization kinetics, forms highly stable phases, and rapidly and uniformly heats precursor materials to a desired temperature. For instance, Chen et al. used microwave-assisted hydrothermal synthesis to prepare a graphene/nickel-oxide composite. NiO particles (20–50 nm) were uniformly dispersed into reduced graphene oxide (rGO) sheets. As an electrode, the material exhibited a high specific capacitance of 617 F g^−1^ at a current density of 1 g^−1^ and a 94.4% capacitance retention after 5000 cycles [127]. Composites prepared by incorporating nickel oxide with other MOs also exhibited a superior electrochemical performance owing to mixed oxidation states, which increased the composites’ pseudocapacitances [128]. Because of its high theoretical specific capacity and different crystal structures and morphologies, Ni(OH)_2_ is another potential SC electrode material [129]. However, Ni(OH)_2_ particles aggregate during continuous charge–discharge cycling, which generates a high charge-transfer resistance at the electrode surface [108]. Therefore, composites consisting of Ni(OH)_2_ combined with other materials, such as graphene [130], CNTs [131], and AC [132], are needed to solve such problems. Yan et al. used a similar rapid low-cost microwave-assisted hydrothermal synthesis method to prepare a flower-like Ni(OH)_2_/graphene-composite SC electrode. An asymmetrically configured SC fabricated using the synthesized Ni(OH)_2_/graphene composite and porous graphene as an anode and cathode, respectively, exhibited a high specific capacitance of 218.4 F g^−1^ and a 94.3% capacitance retention after 3000 cycles [133]. Owing to their rich redox reactions and high electrical conductivities, binary and ternary MOs or hydroxides are very promising for applications to SC electrodes. For example, although Ni–Co layered double hydroxide (LDH) has attracted considerable research interest for applications to SC electrodes [134], pristine Ni–Co LDH exhibits an inferior stability during charge–discharge cycling and a high capacitance loss. However, incorporating nitrogen-doped mesoporous carbon into the electrode is promising for increasing the electron transfer rate and the electrolyte ion diffusion. For example, Xu et al. used microwave-assisted synthesis to prepare nitrogen-doped mesoporous carbon/nickel–cobalt hydroxide microspheres for applications to asymmetrically configured SCs. The device exhibited a specific capacitance of 272.6 F g^−1^ at a discharge current density of 1 Ag^−1^ and a capacitance retention of 87.2% after 10,000 cycles [135]. Cobalt oxides and hydroxides, such as cobaltite (Co_3_O_4_), cobaltous oxide (CoO), and cobaltic oxide (Co_2_O_3_), are also promising SC electrode materials [136]. Among the various structures, nanostructured Co_3_O_4_ is the most widely explored stable structure because both Co^2+^ and Co^3+^ are stable. As an SC electrode material, nanostructured Co_3_O_4_ exhibits a high redox activity, large theoretical capacitance (3560 F g^−1^), low cost, and good reversibility [137]. Microwave-assisted hydrothermal synthesis can be used to effectively control the particle size, and synthesizing uniform porous nanoparticles is important for improving the overall electrode electrochemical performance [138]. Vijaykumar et al. used microwave-assisted synthesis to rapidly prepare Co_3_O_4_ nanoparticles. An electrode fabricated using the nanoparticles microwaved for only 5 min exhibited a high specific capacitance of 519 F g^−1^ [139]. Such a rapid synthesis technique is required for industrially producing SC electrodes. Xu et al. used microwave-assisted synthesis to prepare a Co/rGO-based nanocomposite, as shown in Figure 5a–e. Compared to electrodes fabricated using individual Co and rGO components, the electrode fabricated using the nanocomposite material microwaved for 15 min exhibited an improved specific capacitance of 370 F g^−1^. Additionally, the nanocomposite electrode retained 92.3% of its initial capacitance after 2000 continuous charge–discharge cycles at a current density of 2 A g^−1^ [140]. 

Kumar et al. used cobalt oxides, rGO, and microwave-assisted hydrothermal synthesis to inexpensively and rapidly prepare a Co_3_O_4_/CoO/rGO nanocomposite in a single step for applications to hybrid SC electrodes [141]. Although electrodes fabricated using binary and ternary TMOs exhibit an improved electrochemical performance and reversibility compared to electrodes fabricated using their unitary counterparts, they still exhibit low electrical conductivities, poor cyclic stabilities, and few electroactive sites. However, such drawbacks can be overcome by adding carbon-based conducting materials, such as rGO, to the electrodes’ materials. In addition to an electrode’s material itself, synthesis technique is equally important because it enables electrode materials to be synthesized in a cost-effective, facile, and rapid manner. For instance, Kumar et al. recently used microwave-assisted hydrothermal synthesis to rapidly prepare a honeycomb-shaped NiO/Co_3_O_4_/rGO composite electrode material exhibiting a unique honeycomb-like morphology. The electrode was fabricated using the material and scanned at 20 mV s^−1^, and it exhibited a high specific capacitance of 910 F g^−1^ and a good capacitance retention of 89.9% after 2000 cycles. The improved electrode’s electrochemical performance was attributed to the honeycomb-like morphology and the increased composite conductivity [142]. Vanadium forms TMOs that exhibit multiple oxidation states, including 5+, 4+, 3+, and 2+. To date, V_2_O_5_, V_2_O_3_, and VO_2_ have been discovered [143]. Among them, vanadium pentoxide (V_2_O_5_) is the most studied stable vanadium-based TMO applied to SC electrodes [144].

A good SC electrode material exhibits a high redox activity, a good conductivity, and a large specific surface area. Additionally, a rapid and effective synthesis technique is required. Because electrode materials are rapidly and uniformly heated, microwave-assisted hydrothermal synthesis is very promising for synthesizing vanadium-oxide-based SC electrode materials. Although the layered structure, natural abundance, and high theoretical capacitance of V_2_O_5_ are well known, it exhibits a poor structural stability and electrical conductivity. Thus, the V_2_O_5_ conductive matrix must be investigated to fabricate SC electrodes exhibiting a good electrochemical performance. Toward that objective, several studies have been conducted. For example, Ramados et al. used an inexpensive, environmentally friendly microwave-assisted synthesis technique to rapidly prepare an rGO/V_2_O_5_ composite electrode material. The electrode was fabricated using the composite material and then scanned at 5 mV s^−1^, and it exhibited a good electrochemical performance, including a specific capacitance of 250 F g^−1^ and a high capacitance retention of 95% after 5000 charge–discharge cycles. The enhanced electrode’s electrochemical performance was attributed to the synergistic contributions of both components [145]. Doping V_2_O_5_ with other MOs is another strategy for improving the electrode’s electrochemical performance. For instance, Lathe et al. used microwave-assisted synthesis to prepare pure-V_2_O_5_ and MnO_2_/V_2_O_5_-nanocomposite SC-electrode materials and thoroughly studied the effects of different synthesis parameters on the materials and electrochemical performance of the electrodes fabricated using the prepared materials. The synthesized MnO_2_/V_2_O_5_ nanocomposite was microwaved for 10 min and exhibited an average crystallite size of 17 nm. When 6% MnO_2_ was added to the V_2_O_5_, the specific capacitance of the electrode fabricated using the MnO_2_-doped V_2_O_5_ increased from 150 to 570 F g^−1^ in an aqueous KOH electrolyte. Microwave-assisted synthesis aided a rapid and controlled MnO_2_/V_2_O_5_-nanocomposite particle growth [146]. These studies investigated the novelty, applications, and critical aspects of microwave-assisted synthesis for preparing MOs and composite-based SC electrode materials. Microwave-assisted synthesis shortens the chemical reaction times from several hours to a few minutes, is inexpensive, uniformly heats materials, and reduces energy consumption. Compared with traditional heating methods, microwave heating homogenizes nucleation and accelerates crystallization. Furthermore, microwave-assisted synthesis can be effectively combined with other techniques, such as sol–gel and hydrothermal syntheses.

## 4. Chemical-Bath Deposition

**Figure 6:**
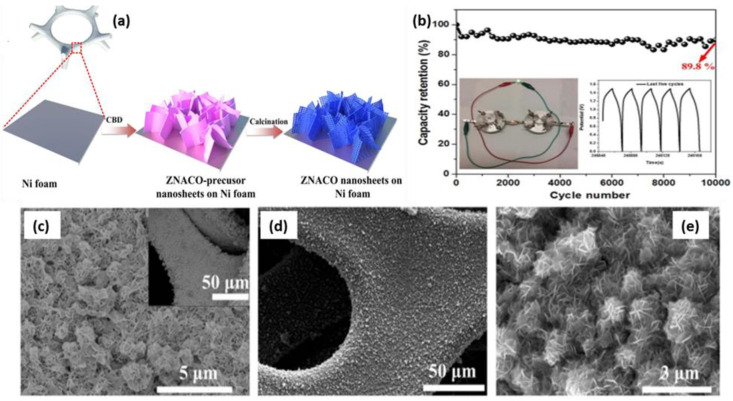
(**a**) Schematic illustrating mechanism of ZNACO nanosheet formation on Ni foam for applications to binder-free electrodes. (**b**) Charge–discharge profiles of synthesized materials and (**c**–**e**) SEM images of the ZNACO nanosheet formation on Ni foam (reprinted with permission from Ref. [149]. Copyright 2016 Elsevier).

CBD is a simple and inexpensive technique for directly depositing semiconducting materials, such as MOs and metal hydroxides, on conducting substrates because it does not require high temperatures or an external electrical source [147]. Compared with hydrothermal and solvothermal syntheses, CBD enables the industrial production of the materials. During CBD, a synthesized material is precipitated from a precursor solution to a substrate surface. In CBD, substrates are dipped into a basic solution composed of precursor materials, such as metal ions, a basic medium, and a chelating agent, to release the metal ions into the solution. CBD enables a film’s thickness and composition to be controlled by adjusting a solution’s pH, temperature, and reagent concentration [112]. Owing to their nontoxicity, natural abundance, high electrochemical activity, and good electrical conductivity, zinc oxides [126] and their composites have recently sparked considerable research interest for applications to SC-electrode materials. For instance, Chebrolu et al. used simple, cost-effective CBD to synthesize ZnO-based composites to investigate the electrochemical performance of ZnO-nanowire-, ZnO/CuO-nanowire-array-, ZnO/NiO-nanosheet-, ZnO/FeO-nanocrystal-, and ZnO/PbO-nanotube-based binder-free electrodes. The synthesized materials were grown directly on a conducting substrate (i.e., Ni foam). The ZnO/NiO nanosheets exhibited the highest pseudocapacitive specific capacitance of 1248 F g^−1^ and the best cycling stability (79%) of all the electrodes after 3000 cycles. The simple CBD method and the synergistic contributions of the *n*-type ZnO and *p*-type NiO improved the electrochemical performance of the electrode [148]. However, the electrode materials exhibited low energy densities, which limited their industrial applications to advanced multifunctional SCs. Using hybrid SCs is an effective strategy for overcoming low-energy-density limitations, and binder-free electrodes improve a hybrid SC’s working performance. However, inexpensively producing these materials is a formidable challenge. CBD is very advantageous for rationally designing and synthesizing multicomponent-based hierarchical MO-based SC structures. For instance, Zhang et al. used CBD to synthesize self-supported porous quaternary Zn–Ni–Al–Co oxide (ZNACO) nanosheets as hybrid electrode materials for applications in SCs, as shown in Figure 6a–e. At a discharge current density of 1 A g^−1^, a binder-free electrode in a three-electrode configuration exhibited a specific capacitance of 839.2 C g^−1^. An asymmetrically configured device fabricated using ZNACO and AC as a cathode and anode, respectively, produced a high energy density of 72.4 W h kg^−1^ while exhibiting a remarkable power density of 533 W kg^−1^. After 10,000 cycles at a current density of 10 A g^−1^, the hybrid SC device retained 90% of its initial capacitance (Figure 6b). The good capacitive performance was attributed to the unique structural features and synergistic contributions of the four MOs. This study also revealed the potential of applying CBD to synthesize quaternary hybrid electrode materials for applications in SCs [149]. 

Because of their vast natural abundance, low cost, nontoxicity, and high theoretical capacity (670 mA h g^−1^), copper oxides and hydroxides are other potential electrode materials for applications in SCs. However, after numerous continuous charge–discharge cycles, pristine copper oxide electrodes exhibit poor structural stabilities. Efforts to overcome these challenges have included developing hybrid nanocomposites exhibiting different crystal structures. Additionally, CBD has been investigated for industrially synthesizing electrode materials. CBD has proven to be well suited for synthesizing copper oxides for applications to SC electrodes because it is simple and cost-effective and enables the electrode material’s crystallinity to be controlled. For example, Patil et al. optimized the CBD synthesis temperature to prepare hybrid CuO/(OH)_2_ thin films exhibiting different morphologies for applications to SC electrodes. The electrochemical performance of electrodes was influenced by the crystal structures and morphologies of the thin films synthesized at different temperatures. The morphologies changed from nanobricks to nanoleaves and then nanobuds with an increasing CBD temperature, and a nanobrick-based electrode exhibited the highest specific capacitance of 340 F g^−1^ at an areal current density of 1 mA cm^−2^ [150]. Lokhande et al. used CBD to synthesize rose-like nanostructured CuO and subsequently used the CuO to fabricate a binder-free electrode. A unique nanostructural morphology and improved electrode electrochemical performance were attributed to the use of Triton^®^ X-100 and a simple, rapid, and binder-free CBD synthesis. At a high discharge current density of 30 A g^−1^, the electrode exhibited a specific capacitance of 550 F g^−1^ [151]. To improve the energy storage properties of SCs, electrode materials must exhibit high energy densities and superior electrical conductivities. Researchers have developed effective strategies, such as using multicomponent nanostructured materials, for increasing the electrode material’s energy density. Using thermal oxidation and CBD, Xu et al. prepared core–shell Ni(OH)_2_@CuO nanowires as binder-free electrode materials on 3D copper foam. The pristine CuO nanowires fabricated on the 3D copper foam exhibited an average specific surface area of 138.2 m^2^ g^−1^. The Ni(OH)_2_ nanoflake shell growth increased the number of electroactive sites available for redox reactions, and the resulting electrode exhibited an areal capacitance of 1.625 F cm^−2^ at a current density of 3 mA cm^−2^. An SC device fabricated using Ni(OH)_2_@CuO and AC as a cathode and anode, respectively, exhibited a high energy density of 58.59 W h kg^−1^ at a power density of 686.45 W kg^−1^ [152]. Owing to their good electrical conductivities, high pseudocapacities, and chemical stabilities, binary MOs, such as MnCo_2_O_4_, ZnCo_2_O_4_, and NiCo_2_O_4_, are promising SC-electrode materials [153,154,155]. Owing to its high energy density, long cycling life, and high power density, MoNiO_4_ has recently attracted considerable research attention as a potential SC-electrode material. Additionally, CBD has been used to improve MoNiO_4_ synthesis because CBD is a low-cost, efficient, rapid, and simple synthesis technique and enables binder-free electrodes to be fabricated. For instance, Kumar et al. used CBD and then calcination to synthesize MoNiO_4_ flower-like nanostructures. A binder-free electrode fabricated using the material exhibited a high specific capacitance of 1140 F g^−1^ at a discharge current density of 2 A g^−1^, high energy density of 64.2 W h kg^−1^ at a power density of 1750 W kg^−1^, and remarkable stability of 97.8% after 3000 cycles. A flower-like morphology and numerous electrochemical sites enabled the deep and smooth penetration of electrolyte ions into the electrode’s surface pores [156]. Waghmode et al. recently used CBD and various urea concentrations to fabricate NiCo_2_O_4_ thin-film-based SC materials exhibiting different morphologies. Furthermore, CBD facilitated the growth of NiCo_2_O_4_ nanoflowers exhibiting nanorod-like thin films on a stainless-steel substrate. The hybrid morphology promoted electronic conductivity and a low ionic diffusion resistance. An electrode fabricated using the NiCo_2_O_4_ thin film synthesized using an optimized urea concentration (2 M) exhibited a specific capacitance of 702 F g^−1^. Additionally, the fabricated flexible solid-state SC device exhibited a specific capacitance of 132 F g^−1^ and an energy density of 18.52 W h kg^−1^ at a power density of 3.13 kW kg^−1^ [157]. Anitha et al. used CBD to prepare a binder-free PbMoO_4_/CdMoO_4_-based composite electrode material on Ni foam and evaluated the electrode’s electrochemical performance. The PdMoO_4_/CdMoO_4_-based composite electrode’s electrochemical performance surpassed that of PbMoO_4_- and CdMoO_4_-nanosheet-based electrode counterparts. In a three-electrode configuration, the composite-material-based electrode exhibited a specific capacitance of 1840.32 F g^−1^ at a discharge current density of 1 A g^−1^. The improved electrode’s electrochemical performance was attributed to the electrolyte ions smoothly, gradually, and deeply penetrating the active material pores, which enabled pseudocapacitive reactions [158].

Furthermore, current collectors (CCs) considerably impact the electrochemical performance of an SC device. A good CC should be inexpensive and exhibit a large 3D surface area and a high conductivity. CBD is more advantageous than electrodeposition because it can be used to grow electrode materials on both conducting and nonconducting substrates. Furthermore, CBD has gained popularity because it is suitable for inexpensively and industrially producing electrode materials. Moreover, CCs greatly improve an SC device’s electrochemical performance. For example, Alhebshi et al. drop-cast slurry onto a substrate to synthesize Ni(OH)_2_ flakes and then deposited them onto carbon microfibers to fabricate binder-free thin-film electrodes. Surprisingly, the electrode fabricated using the binder-free Ni(OH)_2_ nanoflakes exhibited five times the specific capacitance of the slurry-coated Ni(OH)_2_ electrode. This study revealed the importance of both the 3D CC structure and the CBD-fabricated binder-free electrode for enhancing the SC electrode’s electrochemical performance. Furthermore, the binder-free and slurry-coated electrodes exhibited 34 and 62% capacitance drops after 10,000 cycles, respectively, confirming that a binder-free electrode considerably stabilizes an SC working electrode [159].

## 5. Atomic-Layer Deposition

**Figure 7:**
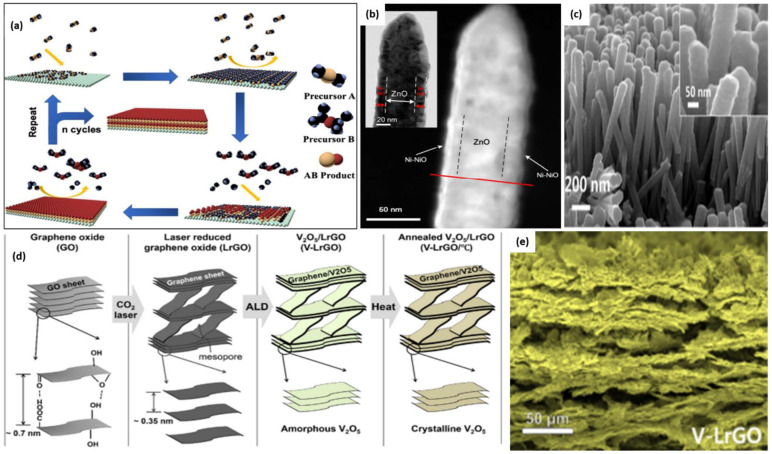
(**a**) Schematic of ALD steps in AB binary reaction sequence (reproduced with permission [168]). (**b**) TEM image of Z-N 800 and (**c**) SEM image of Ti/ZnO NW/Ni–NiO fabricated over 1500 ALD cycles (reprinted with permission from Ref. [169]. Copyright 2018 American Chemical Society). (**d**) Schematic illustrating formation of amorphous and crystalline V_2_O_5_ coated on rGO and (**e**) SEM image of V–rGO fabricated over 300 ALD cycles (reprinted with permission from Ref. [177]. Copyright 2020 Elsevier).

ALD is a multistep chemical-vapor deposition wherein atomically thin films are grown on conducting substrates [160,161]. ALD is important for the cost-effective fabrication of large specific surface-area materials for applications to a wide range of energy-storage devices and optoelectronics [162], nanogenerators [163], LIBs [164], photovoltaics [165], microelectronics [166], and SCs [167]. In ALD, because films are grown on substrates through binary chain reactions, a film’s thickness, morphology, and chemical properties are atomically controlled. As shown in Figure 7a [168], ALD produces highly pure conformal crack- and pinhole-free films on substrates. Compared to conventional ALD, plasma-enhanced ALD (PEALD) is more advanced because of its high reactivity, which reduces deposition temperatures and thus increases deposition rates without compromising the uniformity or quality of a grown film. Various materials can also be used as active precursors for thin-film depositions (Figure 7b,c) [169]. The critical parameters on which an SC electrode’s electrochemical performance depends are a large 3D surface area, good electrical conductivity, and smooth and rapid electrolyte ion diffusion. To achieve such qualities, active electrode materials’ morphologies, dimensions, and uniformities must be precisely controlled. Furthermore, high-rate performance and long-term cycling stability are critical parameters in determining the EC performance [170]. The chemical degradation of active electrode materials during continuous charge–discharge cycles is a major limiter of the electrode cycling performance. Active material degradation forms a resistive layer on the electrode surface and, consequently, hinders electrolyte ions from smoothly and rapidly diffusing. However, ALD offers various advantages for growing active electrode materials for fabricating next-generation SCs. ALD is utilized to improve SC substrates, anodes, cathodes, electrolytes, and separators at the micro and nano scales. Because of their natural abundance, high pseudocapacities, and remarkable theoretical specific capacitances, MOs and their composites are very suitable for applications to SC electrode materials [12,27]. However, poor electrochemical stabilities due to active materials’ degradation during continuous cycling are major limitations. To overcome these drawbacks, ALD uses promising materials to prevent side reactions, thereby preventing electrode/electrolyte interface formations. For example, Chodankar et al. used ALD to deposit a thin NiO layer on NiCo_2_O_4_ nanowires to provide stability during continuous cycling. A core–shell NCO–NiO nanowire grown on a carbon cloth exhibited an outstanding specific capacitance of 2439 F g^−1^ and cycling stability of 94.2% after 20,000 charge–discharge cycles. The NCO/NiO-core–shell-based SC device exhibited an energy density of 72.32 W h kg^−1^ [171]. Because applying ALD to directly deposit active materials on various nanostructured templates has attracted much research attention, highly conformal films enable a controlled ion intercalation and deintercalation deep into the electrode’s surface, resulting in a high capacity. CNTs, graphene, metal/MOs, and organic materials are nanostructured templates on which thin films can be grown using ALD [168]. Vanadium oxides also exhibit low conductivities and poor electrochemical stabilities. Using ALD, Boukhalfa et al. grew a 0.1-nm-thick V_2_O_5_ thin film on conducting CNTs over 500 ALD cycles. Even after 500 ALD cycles, the V_2_O_5_-coated CNTs exhibited cluster- and pinhole-free uniform/conformal coatings. A multiwalled carbon nanotube (MWCNT)–V_2_O_5_ electrode exhibited a specific capacitance of up to 1550 F g^−1^ and a high cycling stability [172]. Because of its large specific surface area, high conductivity, and double-layer capacitance charge storage, graphene has also been used as an MO-growth template [173,174]. Because of its low cost, natural abundance, high electrochemical stability, and environmental friendliness, TiO_2_ is a very promising SC electrode material [175]. Sun et al. provided a good example of coating a graphene (template) surface with TiO_2_. Because the graphene surface is inert, the TiO_2_ nanoparticles nucleated and were uniformly distributed on the graphene surface. A 100-ALD-cycle coating produced 10-nm-thick TiO_2_ thin films on the graphene surface, and a TiO_2_/graphene-based electrode scanned at 10 mV s^−1^ exhibited a specific capacitance of 84 F g^−1^. The uniformly coated film exhibited numerous electroactive sites, which enabled pseudocapacitance [176]. An electrode fabricated using the V_2_O_5_-ALD-coated rGO exhibited an improved electrochemical performance compared to a pristine rGO-based electrode. The resulting amorphous V_2_O_5_ films exhibited many surface defects, disorders, and high porosities (Figure 7d,e). Pristine-rGO- and V_2_O_5_/rGO film-based SCs exhibited specific capacitances of 50 and 189 F g^−1^, respectively, at current densities of 1 A g^−1^. Additionally, ALD enhanced the electrode’s cycling performance with a capacitance retention of 80% after 10,000 cycles [177]. Film-coating thicknesses considerably impacted the SC devices’ electrochemical performance, as demonstrated by Yu et al. A NiO thin-film-based NiO/CNT SC deposited over 200 ALD cycles exhibited the best electrochemical performance (e.g., a specific capacitance of 622 F g^−1^ at a current density of 2 A g^−1^ and a 74% capacitance retention at a discharge current density of 50 A g^−1^). Ozone (O_3_) was used as an oxygen source to deposit a uniform, precise, conductive NiO film at 0.2 Å cycle^−1^. Compared to using H_2_O as an oxidant, which does not react with CNTs because they are hydrophobic, using O_3_ improved the film’s growth by causing NiO to grow at defect and CNT impurity sites. Consequently, NiO was uniformly coated on the CNT surface [178]. According to SC research, the larger the electroactive reaction area of an electrode, the greater the penetration and contact of the electrolyte ions with the active material. By introducing a sacrificial layer beneath the active material layer, ALD aids in generating a disconnected core–shell or hollow nanomorphology. Ample open space also reduces the magnitude of stress–strain generated during continuous charge–discharge cycling, thereby promoting a long-term cycling stability. Guan et al. provided a good example of the use of a sacrificial layer. An Al_2_O_3_ sacrificial layer was covered with CoO nanorods and NiO nanowalls. Then, ALD was used to coat TiO_2_ layers on the Al_2_O_3_ layer, which was subsequently removed by conducting etching with a KOH solution, revealing TiO_2_-shell-capped CoO nanorods and NiO nanowalls. The SC device fabricated using this unique hollow nanoarchitecture exhibited a two- to four-fold-higher specific capacitance and a solid core–shell structure. The enhanced electrochemical performance of the SC device was attributed to the short ion diffusion pathways and large specific surface area of the electrode material [179]. The limited surface-area deposition prevented the active electrode material from being completely utilized because the electrolyte ions could not deeply penetrate the electrode surface. This limitation can be overcome by using ALD because ALD purge cycles enable gaseous precursor molecules to controllably nucleate and grow in the nanospace. This infiltration improves the active electrode material functionalization. For example, Lee et al. used ZnO ALD to incorporate a trace of zinc deep in the rGO interlayer spacing and subsequently irradiated the functionalized ZnO-coated rGO surface with a laser to vaporize the ZnO into Zn through the reaction C(s) + ZnO(s) → Cʹ(s) + Zn(g) + CO (g). Although the resulting rGO was functionalized, it contained defects, such as wrinkles and cracks. The ALD ZnO infiltration increased the functionalized-rGO-based electrode’s electrochemical performance by approximately four times compared to the pristine-rGO-based electrode counterpart [180].

Owing to their hydrophilicities, large specific surface areas, high conductivities, and pseudocapacitive charge-storage mechanisms, MXenes (2D materials) have recently attracted considerable research interest for applications to SCs [181,182]. Surprisingly, compared to an SC device fabricated using a pristine MXene (Ti_3_C_2_T_x_), an SC device fabricated using an MXene surface-functionalized using pseudocapacitive materials exhibited a markedly improved electrochemical performance. For example, ALD was used to prepare a Co–Ni bimetallic oxide core–shell-based pseudocapacitive material, which improved the Ti_3_C_2_T_x_ nanosheet EC performance to 1960 F g^−1^ at a current density of 1 A g^−1^. A capacitance retention of 90.2% after 8000 continuous charge–discharge cycles was attributed to the generation of electroactive sites and the synergistic contributions of CoO_x_ and NiO [183]. Heterojunctions have recently attracted tremendous research interest owing to the development of numerous nanostructured materials, including RuO_2_, TiO_2_, Co_3_O_4_, NiO, SnO_2_, WO_3_, ferrites, and perovskites [184]. SC electrodes fabricated using these materials must be inexpensive and nontoxic and must exhibit large specific surface areas, high electrochemical conductivities, and stability. Heterojunctions fabricated using these materials have also been applied to SC electrodes. For example, using highly precise ALD, Hai et al. synthesized an ultrathin (conformally ~12-nm-thick) WO_3_/TiO_3_ heterojunction. ALD is superior to other synthesis methods because it enables atomically thin films to be deposited approximately one atomic layer at a time, which is important when a material requires a large specific surface area. An SC electrode fabricated using a 12-nm-thick WO_3_/TiO_2_ heterojunction exhibited a specific capacitance of 625.63 F g^−1^ [185].

## 6. Conclusions and Outlook

Because of their high power densities, long cycling lives, rapid charging and discharging, low costs, and remarkable energy densities, SCs are in high demand as electrochemical energy-storage devices. With advancements in nanostructured materials, SC technology is gaining popularity because it can meet the energy storage requirements of advanced multifunctional applications. Various electrode materials, such as carbonaceous materials, conducting polymers, and MOs, have been thoroughly investigated for applications to SC materials. Herein, we reviewed MOs and their composite-based SC electrode materials synthesized using various techniques. Because a low SC energy density is one of the most important challenges to overcome, considerable effort has been made to increase SC energy densities while maintaining high power densities. MOs and their composites can provide higher energy densities than conducting polymers and carbon-based electrode materials and are fascinating because of their low costs, high specific capacitances, good electrical conductivities, and high mechanical and chemical stabilities. The most important factors affecting SC devices’ electrochemical performance are electrode materials exhibiting nanostructured morphologies and pores that can be readily penetrated by electrolyte ions; large specific surface areas, so that electrolyte ions can enhance surface wettabilities; and short ion diffusion pathways and surfaces functionalized with numerous electroactive sites for redox reactions. Because fabricating MO-based electrode materials exhibiting such properties primarily depends on synthesis techniques, not only the active electrode material but also the synthesis technique must be carefully chosen to achieve an excellent electrochemical performance. This review investigated hydrothermal and microwave-assisted syntheses, CBD, and ALD. Hydrothermal synthesis is commonly used to prepare nanostructured films and nanopowders and enables MO-based electrode materials exhibiting wide ranges of morphologies to be industrially produced. However, hydrothermal synthesis requires high temperatures and lengthy operation times. Microwave-assisted synthesis is an electrochemical radiation-based technique for ultrarapidly preparing electrode materials exhibiting unique morphologies. However, electrode materials are expensive to produce using microwave-assisted synthesis and material production rates are low. Although CBD is commonly used for commercially producing nanostructured thin films, it cannot be applied to prepare certain MOs. Because “atomic-layer deposition” refers to the deposition of atomically thick films, ALD is usually used to control and digitally deposit atomically thick thin films directly on substrates. Although ALD enables electrode materials to be industrially produced, it requires extensive experimental conditions and is expensive. This article delves into the critical aspects of using these synthesis techniques for fabricating MO-based SC electrodes and reports the most pertinent electrochemical performance achieved so far using SC devices fabricated using the materials synthesized using with these techniques. Currently, all the reviewed synthesis techniques are promising, and the SC devices fabricated using MO-based electrode materials synthesized using these techniques have exhibited impressive electrochemical performances. However, these synthesis techniques must be improved to nanoengineer existing MOs and composites and fabricate high-performance SC electrodes.

While noteworthy developments have been made in metal-oxide-based electrodes for SCs, there are still a few challenges for the advancement of SCs. There are several challenges in the development of advanced and large-scale supercapacitors, especially in the areas of wearable and flexible electronics. Developing low-temperature, low-carbon, and cost-effective methods of synthesizing these materials is highly desirable. With large-scale production, it is becoming crucial to further modify the material properties of these metal oxides in order to boost their surface areas, active sites, and wettabilities for practical applications of supercapacitors. Adding carbonaceous materials to metal oxides can enhance conductivity by providing extra pseudocapacitances, enhancing surface areas, improving porosities, easing electron and proton conductions, and creating synergistic effects between components. Researchers should aim their efforts at how to obtain raw materials for electrodes at low costs and with no pollution; the use of biomass carbon materials is a suitable example. An emerging area of research to unravel the different roles of modified metal-oxide-based materials involves detailed analyses of the mechanisms involved in the enhanced energy-storage performance of metal oxides based on spectroscopic studies with supportive theoretical research.

## Data Availability

Not applicable.
